# Green Sturgeon Distribution in the Pacific Ocean Estimated from Modeled Oceanographic Features and Migration Behavior

**DOI:** 10.1371/journal.pone.0045852

**Published:** 2012-09-21

**Authors:** David D. Huff, Steven T. Lindley, Brian K. Wells, Fei Chai

**Affiliations:** 1 Fisheries Ecology Division, Southwest Fisheries Science Center, National Oceanic and Atmospheric Administration, Santa Cruz, California, United States of America; 2 Institute of Marine Sciences, University of California Santa Cruz, Santa Cruz, California, United States of America; 3 School of Marine Sciences, University of Maine, Orono, Maine, United States of America; University of Otago, New Zealand

## Abstract

The green sturgeon (*Acipenser medirostris*), which is found in the eastern Pacific Ocean from Baja California to the Bering Sea, tends to be highly migratory, moving long distances among estuaries, spawning rivers, and distant coastal regions. Factors that determine the oceanic distribution of green sturgeon are unclear, but broad-scale physical conditions interacting with migration behavior may play an important role. We estimated the distribution of green sturgeon by modeling species-environment relationships using oceanographic and migration behavior covariates with maximum entropy modeling (MaxEnt) of species geographic distributions. The primary concentration of green sturgeon was estimated from approximately 41–51.5° N latitude in the coastal waters of Washington, Oregon, and Vancouver Island and in the vicinity of San Francisco and Monterey Bays from 36–37° N latitude. Unsuitably cold water temperatures in the far north and energetic efficiencies associated with prevailing water currents may provide the best explanation for the range-wide marine distribution of green sturgeon. Independent trawl records, fisheries observer records, and tagging studies corroborated our findings. However, our model also delineated patchily distributed habitat south of Monterey Bay, though there are few records of green sturgeon from this region. Green sturgeon are likely influenced by countervailing pressures governing their dispersal. They are behaviorally directed to revisit natal freshwater spawning rivers and persistent overwintering grounds in coastal marine habitats, yet they are likely physiologically bounded by abiotic and biotic environmental features. Impacts of human activities on green sturgeon or their habitat in coastal waters, such as bottom-disturbing trawl fisheries, may be minimized through marine spatial planning that makes use of high-quality species distribution information.

## Introduction

Green sturgeon (*Acipenser medirostris*) status is of rising conservation concern, with one distinct population segment of the species listed as threatened under the US Endangered Species Act and the other treated as a species of concern. Canada considers the green sturgeon a species of Special Concern under the Canadian Species at Risk Act [1]. The causes of the decline of green sturgeon are likely manifold and include degradation of freshwater and estuarine habitats [2], blockage of historical habitats by impassable dams [3], and perhaps the past effects of commercial fishing, including bycatch in bottom-trawl fisheries [4]. It is also possible that both the poor status of green sturgeon and the limited understanding of the causes of the green sturgeon’s decline are due in large part to a migratory life history that exposes them to myriad threats in coastal, estuarine, and riverine waters.

In North America, green sturgeon spawn in three rivers between central California and southern Oregon and move among various estuaries, marine waters and natal rivers in a complex migratory cycle that enables them to exploit resources supported by food webs far from their natal spawning grounds [5]. Green sturgeon migration cycles appear to be relatively regular and predictable along defined routes and tend to terminate in winter-time concentration areas off the west coast of Canada where the Subarctic Current bifurcates into the Alaska Current, which runs northwest, and the California Current, which runs southward toward Mexico [4]–[6]. These documented sturgeon overwintering aggregations are sheltered in rocky, high-relief areas less than 200 meters deep and are associated with ephemeral, yet abundant standing stocks of plankton that, in turn, support rich benthic communities [7]–[10].

**Figure 1:**
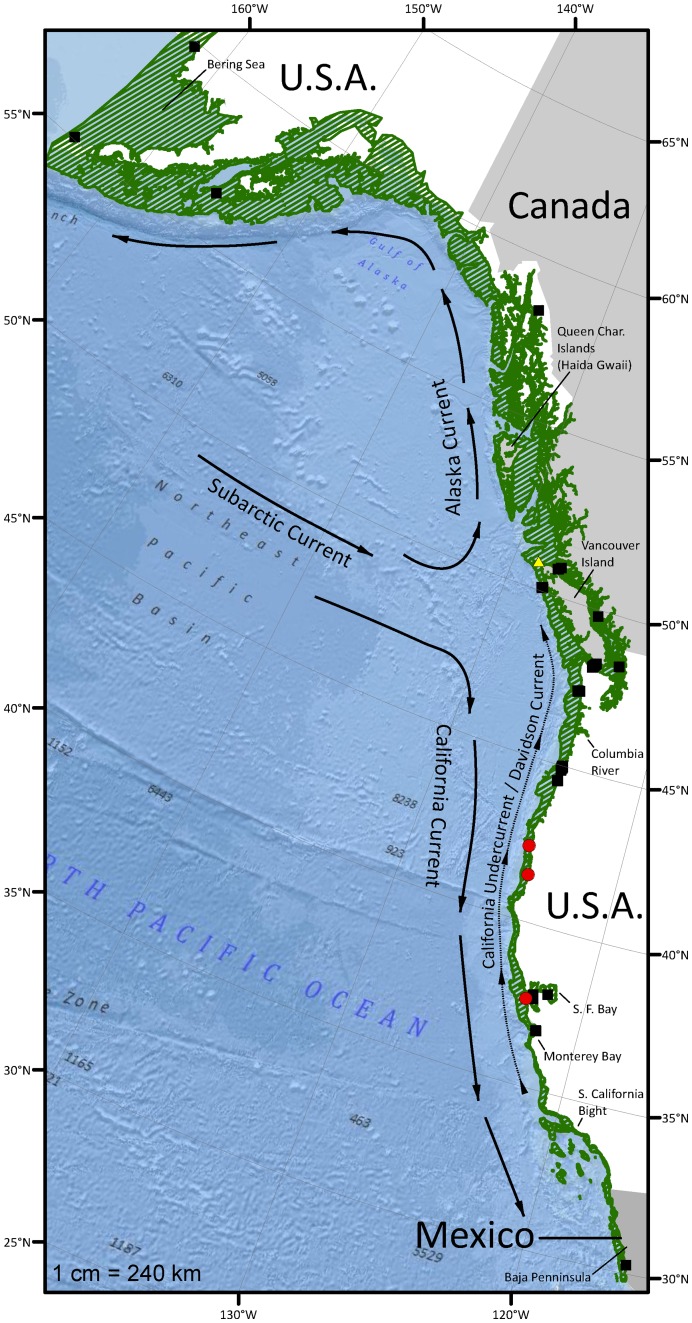
Map of the study area. The green hatched area indicates the geographic range where green sturgeon have been encountered from Baja, California, Mexico to the eastern Bering Sea from shore to the 200 m isobath. Black squares designate the locations of green sturgeon presence records used in this study, red circles denote spawning river mouths, and the yellow triangle indicates the northern overwintering aggregation location [5]. The subarctic current, its bifurcation into the Alaska and California Currents, and the counter current were redrawn from Favorite et al. (1976) and Thomson (1981) [18], [19]. The map is displayed in the Albers Equal Area projection.

However, the broad geographic range where sturgeon have been encountered, from Baja, California to the Bering Sea, extends beyond known northern overwintering grounds and southern spawning rivers (Figure 1) [11], [12]. Limiting factors, such as water temperature, dissolved oxygen, or bottom currents may physiologically bound suitable habitat for green sturgeon within portions of their range seasonally and may vary on a yearly basis depending on the intensity of currents and other factors in warm versus cool years [6], [13]. In contrast to what is known about other migratory fishes such as Pacific salmonids, the physiological ecology and niche breadth of adult green sturgeon are poorly understood and may differ importantly from those of the more frequently studied, but less marine-oriented, sympatric white sturgeon (*Acipenser transmontanus*) [14].

Current or planned human activities that might impact green sturgeon or their habitat in coastal waters include bottom-disturbing trawl fisheries, no-take marine reserves, wave and tidal energy projects, offshore aquaculture facilities, and dredge disposal. Marine spatial planning is envisioned to help site such projects, and better information on the seasonal distribution of green sturgeon is valuable to such efforts [2]. Modeling the distribution of broad-ranged, demersal, marine species has been challenging due to a deficit of consistent environmental data needed to characterize areas with known occurrence versus those that are merely within the limits of potential dispersal. Methodological advances in oceanographic modeling such as Regional Oceanographic Modeling Systems (ROMS) may provide robust and detailed interpolation of oceanographic parameters near the seafloor where they are most relevant to demersal species such as the green sturgeon [15]. These data are often available across entire landscapes and over various time intervals; they may coincide with past survey locations and be used for estimating seasonal variation or future conditions.

We examined the seasonal distribution of green sturgeon throughout their range by modeling potentially complex species-environment relationships using variables derived from oceanographic models, in addition to depth and migration behavior variables, with maximum entropy modeling (MaxEnt) of species geographic distributions [16]. MaxEnt is a statistical method that relates the distribution of habitat characteristics among the sample locations where the species is present to those across the species range. Presence-only distribution models are increasingly being used for fishes and have already been widely applied to many terrestrial species [17]. Our MaxEnt models delineated habitat potential on the basis of the environmental setting in locations and seasons of known use relative to our null distribution model, which is based solely on the maximum depth and latitudinal extent where green sturgeon are known to occur. We describe spatial and temporal patterns in the oceanographic model output and discuss which factors likely determine green sturgeon distribution in the marine environment.

## Materials and Methods

### Study Area

The study area (Figure 1) primarily comprises four oceanographic regions: the eastern Bering Sea, the coastal downwelling domain north of Vancouver Island, BC; the coastal upwelling domain south of Vancouver Island; and a transitional area between the two domains roughly in the vicinity of The Queen Charlotte Islands, BC to the Columbia River [6], [18]. The coastal downwelling domain is characterized by the poleward flowing Alaska Coastal Current, which extends about 40 km off the coast from northern British Columbia and continues around the eastern Gulf of Alaska to Unimak Pass on the Alaskan Peninsula, where it flows at an average speed of about 20 km d^−1^. Nearshore currents are stronger in winter than in summer, and once they turn westward along the coast of the Alaska Peninsula and Aleutian Islands they join the more seaward Alaska Gyre to form a strong boundary current that flows at mean velocities greater than 86 km d^−1^ [19]. The coastal upwelling domain from Baja, California, Mexico to the south coast of British Columbia is defined by a typical summer pattern of upwelling (i.e. Ekman divergence) from about May to September in which cold, subsurface water rises to the surface. The California Current flows equatorward within about 1000 km of the west coast of North America. The California Undercurrent flows poleward and in the winter the nearshore surface expression of this current is often referred to as the Davidson Current. In the summer, surface flow (<200 m) is driven equatorward by persistent winds from the northwest [20], [21]. The transitional area between upwelling and downwelling domains is subject to the somewhat chaotic branching of the Alaska and California currents between 45–50° N and 130–150° W [19]. This is a highly variable, lower-salinity region which is affected by seasonal and inter-annual freshwater discharge from large rivers from north of Vancouver Island to the Columbia River in the south [18]. In the eastern Bearing Sea, our study area lies primarily within the inner shelf domain; interactions there between sea ice, tidal currents, and dilution from coastal rivers drive a highly variable and productive ecosystem [22], [23]. The surface layer in the shallow, eastern Bering Sea is composed primarily of water from the sub-arctic or Alaska Current Systems [18].

### Occurrence Data

We obtained the majority of presence records (192 of 200 records) used in this analysis from coastal tracking arrays that recorded the passage of acoustically-tagged green sturgeon that were collected for previous green sturgeon migration and benthic physical habitat studies. Additional details may be found in Huff et al. and Lindley et al. [5], [24]. We quantified the number of days present for each tagged sturgeon at each hydrophone by summing the time elapsed between acoustic detections in which at least two sequential detections occurred without intervening detections at another hydrophone. Our acoustic tag dataset contained 148 sturgeon at 123 hydrophones for a total of 1450 sturgeon detection-days. We then aggregated hydrophone presence records by season (summer = June, July, August; autumn = September, October, November; winter = December, January, February; spring = March, April, May) from January 2004 to November 2006 when a sturgeon was present for at least a day within each season such that there was no more than one presence record per hydrophone within each season.

To improve the spatial representation of occurrence data within the known geographic extent, we supplemented our acoustic dataset with eight presence records from other sources that were associated with a collection month. Three records from 2005 and 2006 in the northernmost portion of the range were documented in Colway and Stevenson (2007) [12]. The electronic dataset, FishBase, provided another four records, one from near the Taku River mouth in southeast Alaska (1959), and three from San Francisco Bay, California (1941, 1953, and 1956) [25]. The southernmost record in our dataset was recorded in December of 2008 near El Socorro, Baja California and was documented in Rosales and Alameda (2009) [11].

### Distribution Model Covariates

We summarized our oceanographic model covariates by season so that they temporally coincided with the occurrence data and could be used to construct a separate model for each season. Model covariates included temperature, dissolved oxygen, northward and eastward currents, depth, distance to spawning rivers (spawning attraction), and distance to overwintering grounds (overwintering attraction). All covariates were converted to uniform grids with a 1 km cell size and an extent defined by the 200 m isobath (the maximum depth green sturgeon are normally found) along the west coast of North America from the most northerly to the most southerly green sturgeon presence records (Figure 1). Spatial data manipulation, tabulation, and interpolation were implemented using ESRI™ ArcMAP® v.10. Depths were derived from the General Bathymetry Chart of the Oceans (GEBCO_08) 30 arc-second, continuous gridded terrain model for the ocean and land [26]. Distance to spawning and wintering grounds for each cell were calculated by first generating a cost surface grid that was based on reclassifying the depth grid so that ocean floor depths in which green sturgeon typically migrate, from 10 to 200 m [4], [5], [24], were given the lowest cost value of 1. Bottom depths of 0–10 m were assigned a cost value of 5 to approximate an intermediate avoidance for very shallow depths, and depths greater than 200 m were assigned a cost value of 10 to represent the greatest avoidance level, since sturgeon are seldom documented deeper than 200 m [4], [5], [24]. We calculated the accumulated “cost”, for each cell, for the shortest route to both the mean location of all spawning rivers (near the mouth of the Eel River, California, USA) and for the shortest route to overwintering grounds north of Vancouver Island, Canada (Figure1) based on the least accumulative cost path over our cost surface grid. The shortest distance between spawning and overwintering grounds is equal to about 5000 of our calculated cost units. In order to accurately represent migration behavior, the overwintering attraction covariate was only used in the autumn and winter models and the spawning attraction covariate was only used in the spring and summer models.

We generated the remaining four model covariate values (temperature, dissolved oxygen, northward (meridional), and eastward (zonal) horizontal currents) from oceanographic models originally developed for the northern Pacific Ocean (described below), from the water column at the seafloor depth. We matched monthly average oceanographic model output values generated for each month from 2004–2006 to the corresponding month and year for each of our occurrence records. Occurrence records outside this time window and randomly chosen background samples (described below) were matched with three-year (2004–2006) seasonal average values. All oceanographic data were interpolated to the same cell size and extent as the depth grid from a 0.125 degree point grid using the Kriging method [27].

### Oceanographic Model Description

The physical model is based on the Regional Oceanic Modeling System (ROMS) in a similar configuration to those generated by Xiu et al. and Xiu and Chai for the Pacific Ocean (45°S–65°N, 99°E–70°W) [28], [29], with a horizontal resolution of 0.125° and 30 vertical layers. The biogeochemical model is based on the Carbon, Si(OH)_4_, Nitrogen Ecosystem (CoSiNE) model including silicate, nitrate and ammonium, two phytoplankton groups, two grazers and two detrital pools [30]. The dissolved oxygen is linked with air-sea oxygen flux and biological production in the euphotic zone, and oxygen consumption is connected with detritus material remineralization processes at depth.

The Pacific ROMS model has been forced with the climatological National Center for Environmental Research/National Center for Atmospheric Research (NCEP/NCAR) reanalysis of air-sea fluxes [31] for several decades in order to reach quasi-equilibrium. The model is then integrated for the period 1991–2010 forced with daily air-sea fluxes of heat and freshwater from the NCEP/NCAR reanalysis [31]. The heat flux is derived from short- and long-wave radiation along with sensible and latent heat fluxes that are calculated using the bulk formula with prescribed air temperature and relative humidity. The fresh-water flux is derived from the prescribed precipitation and evaporation converted from latent heat release. The blended daily sea wind with a resolution of 0.25° [32] is used to calculate the surface wind stress based on the bulk formula of Large and Pond (1982) [33]. Three-day averaged model outputs were saved from 1991 to 2010. This coupled ROMS-CoSiNE model configuration has been used and evaluated with independent observations repeatedly [34]–[38].

### Distribution Modeling

Initially, we generated a simple ocean distribution (i.e. a null model) for green sturgeon by interpolating among occurrence records from various sources [5], [11], [12], [24], [25] from shore to the 200 m isobath, a typical maximum depth based on known seafloor depth preferences, along the West Coast of North America (Figure 1) [4], [24]. We developed green sturgeon distribution models for each season averaged over three years (2004–2006) with MaxEnt software (MaxEnt v3.3.3 k) [16]. MaxEnt models are probability density comparisons in covariate space [39]. If the conditional density of the covariates at the presence sites and the marginal density of the covariates across the study area are known, then knowledge of the species prevalence will allow calculation of the conditional probability of occurrence (see Elith et al. 2011; Equation 1) [39]. Because prevalence data do not exist for presence-only scenarios, MaxEnt estimates the ratio of the covariate conditional density at presence sites to the marginal covariate density across the study area. For our model, the marginal covariate density was obtained from “pseudo-absences” consisting of 8000 background points that were randomly chosen from the extent of the study region, which is represented by the green hatched area in Figure 1 [40]. MaxEnt fits models using an expanded set of transformations of the original covariates (termed features), using linear, product, quadratic, hinge, threshold, and categorical functions, which allows a great deal of flexibility for modeling non-linear species-habitat responses. MaxEnt selects features with a penalized maximum likelihood model that balances model fit and complexity and produces a smoothed distribution in which an error bound is calculated for each feature based on an adjustable regularization parameter. Conceptually, the regularization parameter corresponds to the product of the width of the standard error interval and a multiplier. We ran several preliminary MaxEnt models with various regularization values and evaluated the subsequent response curves and model fits. As we increased the regularization, model-fit statistics (see description below) became worse, but the generality of the response curves increased (i.e. the response curves were less specifically fit to individual data points). We set the regularization value to three rather than the default setting of one because it balanced the response curves generality with satisfactory model performance and helped avoid overfitting the model to the calibration data [17], [40]. The remaining MaxEnt options were set to a convergence threshold of 10^−5^ and 10,000 maximum iterations. See Elith et al. (2011) and Phillips et al. (2006 and 2008) for detailed explanations of species distribution modeling with MaxEnt [16], [39], [40].

We used K-fold (K = 10) cross-validation in which we split the data into equal-sized parts and then iteratively used part of the data to fit the model and a different part to test it [41]. This method of cross-validation was used to help address the residual effects of spatial autocorrelation on model performance evaluation after tabulating the acoustic detection data in monthly occurrences. We constructed receiver operating characteristic curves (ROC), which are trade-off visualizations for specific pairs of performance measures across a range of presence thresholds for the logistic output, to compare model prediction performance for the cross-validation data partitions [42]. Because our model only incorporated presence data, we applied the previous randomly selected pseudo-absences instead of observed absences to construct the ROC and calculate the corresponding area under the curve (AUC) statistic [16]. The AUC is a commonly used test statistic for evaluating ROC in which scores range from 0.5, which indicates a model that provides predictions that are no better than random, to 1, which indicates a model with perfect predictive ability. Scores considered to be “outstanding” are greater than 0.9 [43].

We constructed maps that displayed MaxEnt logistic probability values averaged across four seasons and distribution plots of the probability of presence for background sites versus latitude across the green sturgeon range (30–60° N). We used a binary probability threshold for each season that defined the point of equal sensitivity and specificity based on the model’s omission of presences (test presences) that were withheld from model construction for testing [43], [44]. A probability threshold may also be chosen based on the acceptable number of test sites that would be omitted by a given threshold. We used the equal sensitivity and specificity approach to threshold choice because it is considered an objective approach and produced thresholds that omitted very low numbers of test presences [44], [45]. We deemed this to be an appropriate criterion because for green sturgeon conservation, the potential for false positive results was considered less important than the potential for false absences.

We assessed model covariate contributions by calculating importance values for each covariate (normalized to percentages) that indicate the influence of each covariate on the final model by randomly permuting the covariate values and measuring the resulting decrease in AUC. We verified the covariate importance values by examining the jackknifed improvement in penalized average log-likelihood compared to a null model, termed “gain” (implemented using MaxEnt software). This procedure systematically removes each variable and then creates a model with the remaining variables, in addition to a model with each variable in isolation. Gain is recomputed each time and a comparison is made to a model with all variables included. Finally, we evaluated the divergence between presence and background samples for each seasonally fluctuating covariate with a median test, a version of non-parametric ANOVA that utilizes a contingency table [46].

## Results

### Model Evaluation

Our model evaluation results indicated that MaxEnt models we constructed for all four seasons discriminated suitable green sturgeon habitat satisfactorily. Average area under the curve (AUC) values for all partitions of both training and test data in all four seasons were >0.98 (Table 1), which is considered outstanding model performance [43]. A high level of uniformity among replications was indicated by low estimates of standard deviation among k-fold model replicates (0.01–0.02). Omission rates (false absences) for test records, not used for model calibration, ranged from 6–9% of the presence samples for the four seasonal models based on equal sensitivity and specificity logistic thresholds, which ranged from 0.14–0.19 (Table 1). A binomial test of omission for the null hypothesis that test points are predicted no better than random produced one-sided p-values <0.01 for all seasons and provided further validation that our models were reliable [16]. MaxEnt receiver operating curves and plots of the omission rate for test model runs, as a fraction of background habitat predicted versus the cumulative threshold of suitable habitat, are provided in the supplemental material (Figure S1).

**Table 1:** Model and covariate evaluation.

Statistic	Summer	Autumn	Winter	Spring
Average Training AUC	0.97	0.98	0.98	0.98
Average Test AUC	0.96	0.97	0.98	0.98
Prevalence	0.05	0.04	0.03	0.03
Logistic Threshold	0.17	0.19	0.14	0.16
Test Site Omission Rate	0.09	0.07	0.06	0.07
Temperature Importance	1	6	9	71
Oxygen Importance	7	4	5	7
Eastward Current Importance	1	33	47	12
Northward Current Importance	1	2	1	2
Depth Importance	4	5	4	2
Attraction Importance	87	50	34	5

10-fold cross-validation was used with 180 training samples and 20 test samples for each fold. Prevalence is the average logistic output for all background sites and the logistic threshold is based on equal test sensitivity and specificity for test site omissions. Importance values for each covariate (normalized to percentages) indicate the contribution of each covariate to the final model by randomly permuting the covariate values and measuring the resulting decrease in AUC.

### Response Curves and Importance of Covariates

**Figure 2:**
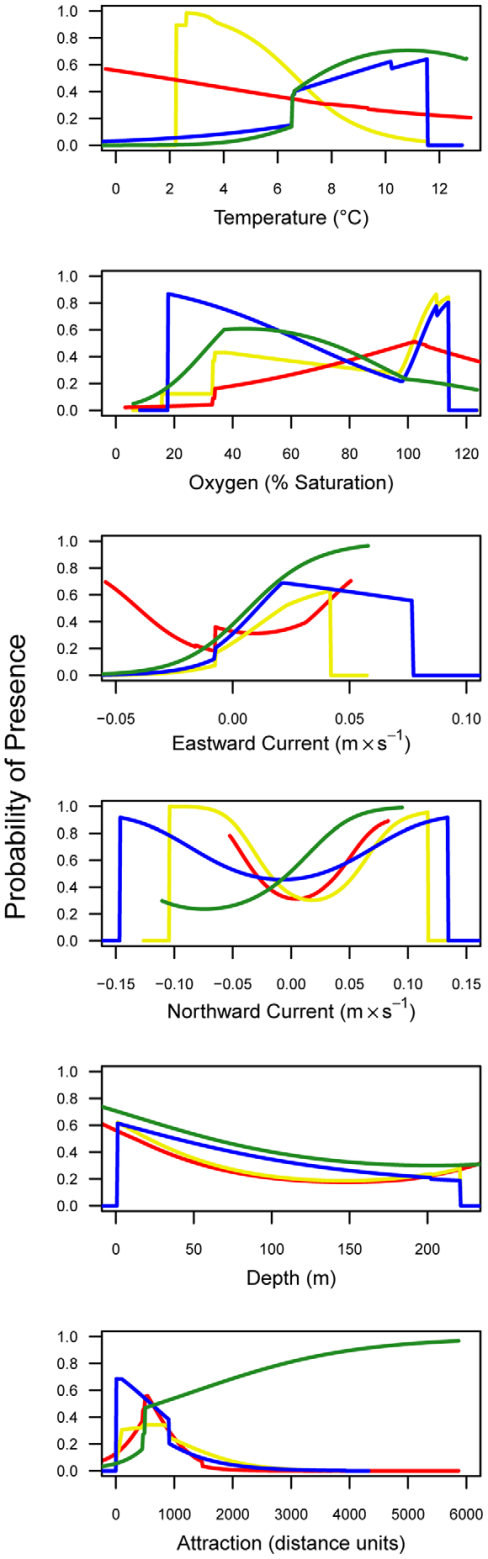
Response curves. Plots depict the seasonal (red = summer, yellow = autumn, blue = winter, green = spring) marginal response of green sturgeon to each of the six model covariates while keeping all other environmental covariates at their average sample values. The axes indicate logistic output (i.e. probability of presence from 0 to 1). For the attraction covariates, the distance between spawning and overwintering grounds is equal to about 5000 cost units. Overwintering attraction is used in the autumn and winter when zero represents the overwintering location and spawning attraction, used in the summer and spring, indicates the mean location of the three spawning river mouths.

Attraction to spawning or overwintering grounds was the most important covariate overall (average permutation importance = 44%) and was the first- or second- most important variable in every season except spring, in which it had relatively little importance (Table 1). The values of depth and (overwintering and spawning) attraction covariates were fixed based on their geographic location and did not vary seasonally. Depth was of relatively low importance in every season, but was of greatest importance in autumn (5%). All seasonal response curves for depth indicated the probability of sturgeon present decreased with greater depths (Figure 2). Overwintering attraction had high importance in autumn (50%) and winter (34%), and spawning attraction was important in summer (87%). Probability of presence was greatest at low distances from spawning or overwintering grounds in the summer, autumn and winter, whereas the relatively uninfluential spring response to spawning attraction (Table 1) showed the greatest probability of presence farthest from the spawning grounds (Figure 2).

**Figure 3:**
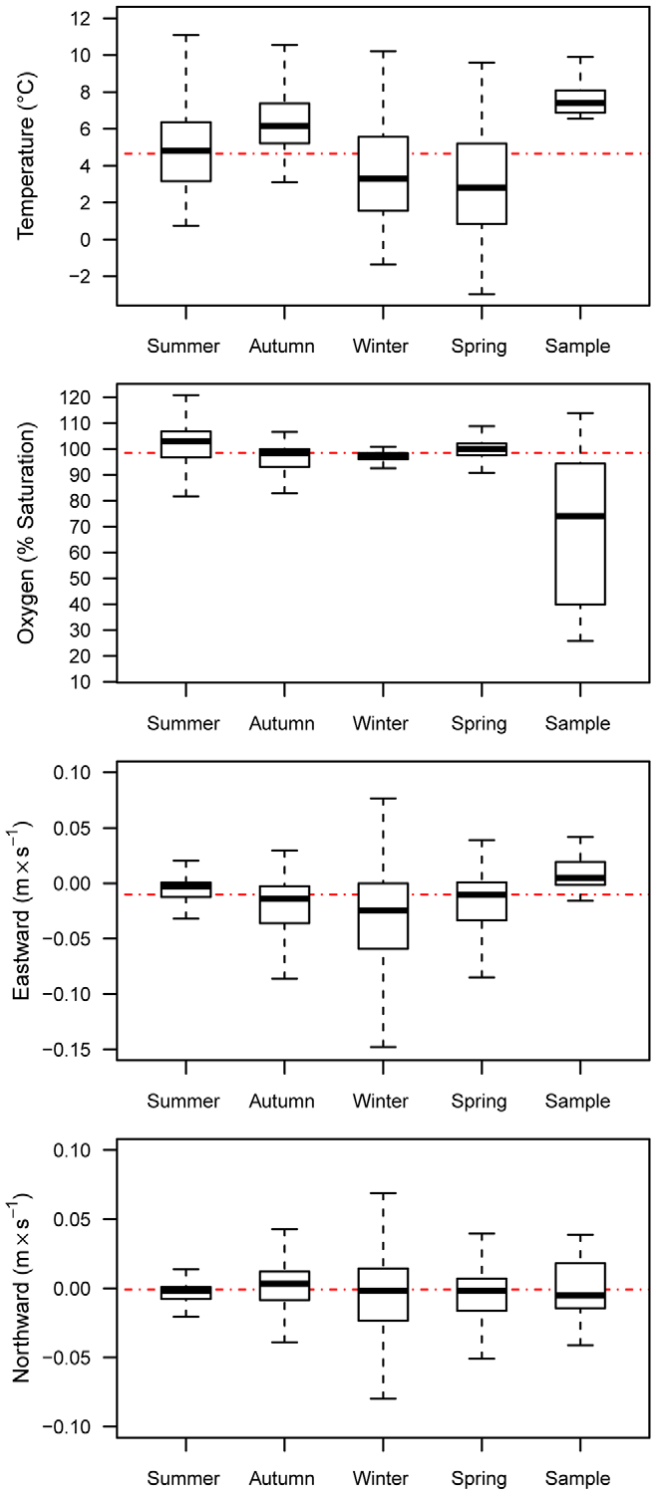
Boxplots of seasonally varying background and observed covariates. Median (closed bar), 1^st^ and 3^rd^ quartile (rectangle) and range (whisker) for background samples by season (first four boxplots), and for presence samples (last boxplot on the right). The red dashed line in each panel represents the overall median.

The northward current covariate was not influential in any season, whereas the eastward current covariate was most important in the winter (47%), followed by autumn (33%), and had less influence in the summer and spring (Table 1). During autumn and winter, when eastward current (negative values indicate westward flow) was most influential for the distribution model, the distance (attraction) to overwintering was also highly influential. Increasing eastward current was associated with greater sturgeon probability of presence in autumn and winter (Figure 2). The median value of the eastward current for presence samples was greater than the median seasonal background sample values (median test; overall median = −0.01 m sec^−1^, P<0.01), whereas none of the northward current values were significantly different from one another (Figure 3). Evaluation of the response curves generated from the MaxEnt models (Figure 2) illustrated the tendency for a greater green sturgeon presence at modeled bottom temperatures from about 6 to 12°C in spring and winter. Temperatures for green sturgeon samples were distributed warmer than the overall median (median test; overall median = 4.7°C, P<0.01) and relative to background samples in all seasons (Figure 3). Dissolved oxygen was more important in summer and spring (7% in both seasons; Table 1). The response curves for dissolved oxygen indicated that above a certain threshold (∼20% saturation in winter and spring and ∼30% in summer and autumn) there was a greater green sturgeon probability of presence (Figure 2). The distribution of dissolved oxygen values among the presence samples was lower than in the background samples for all seasons (median test; overall median = 97% saturation, P<0.01). Relative covariate importance results based on the jackknifed improvement in penalized average log-likelihood compared to a null model (Figure S2) were largely consistent with permutation results (Table 1).

### Predicted Distribution

**Figure 4:**
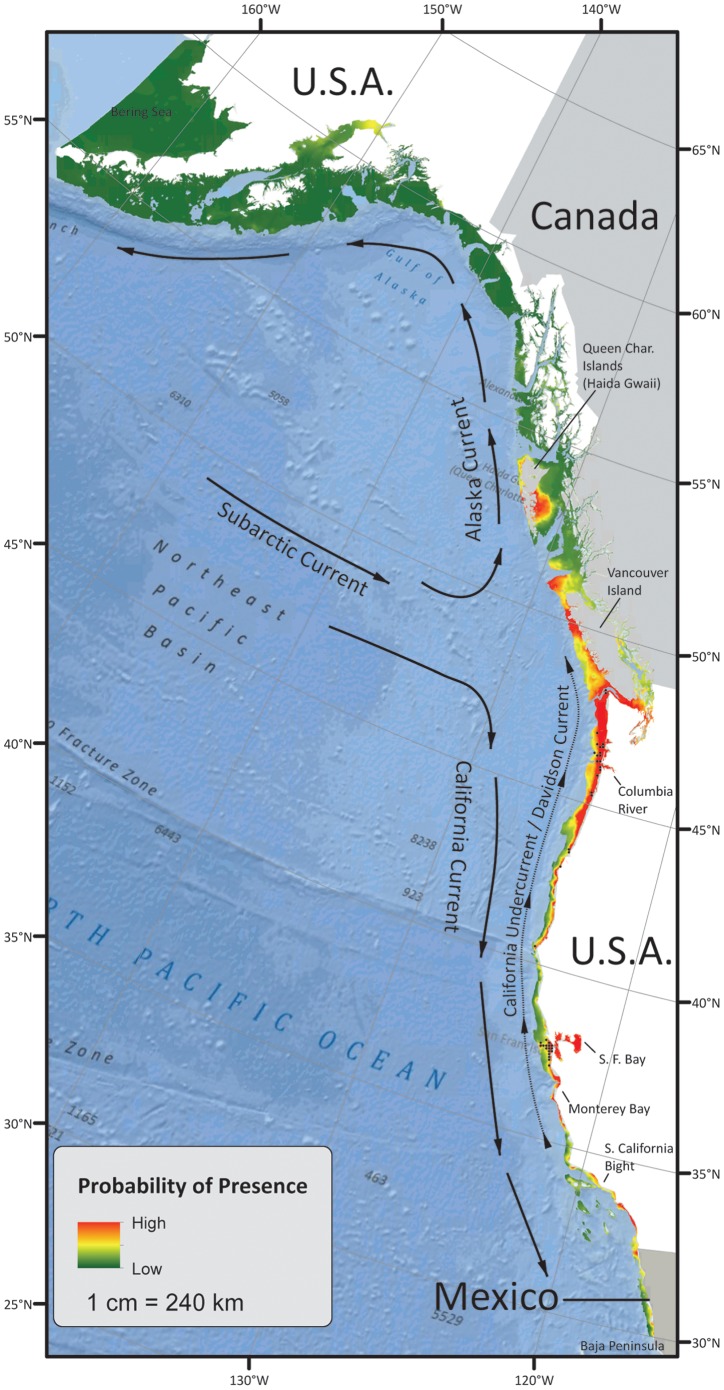
Predicted green sturgeon distribution. Probability of presence interpolated from MaxEnt background predictions averaged across all seasons (maximum probability = 0.7, minimum probability = 0). Individual season interpolations are displayed in Figure S4. Black dots indicate green sturgeon presence aggregated per 10 km^2^ from the West Coast Groundfish Observer Program (see Figure S3). The background layer is from the ESRI Ocean Basemap (2012). The map is displayed in the Albers Equal Area projection.

**Figure 5:**
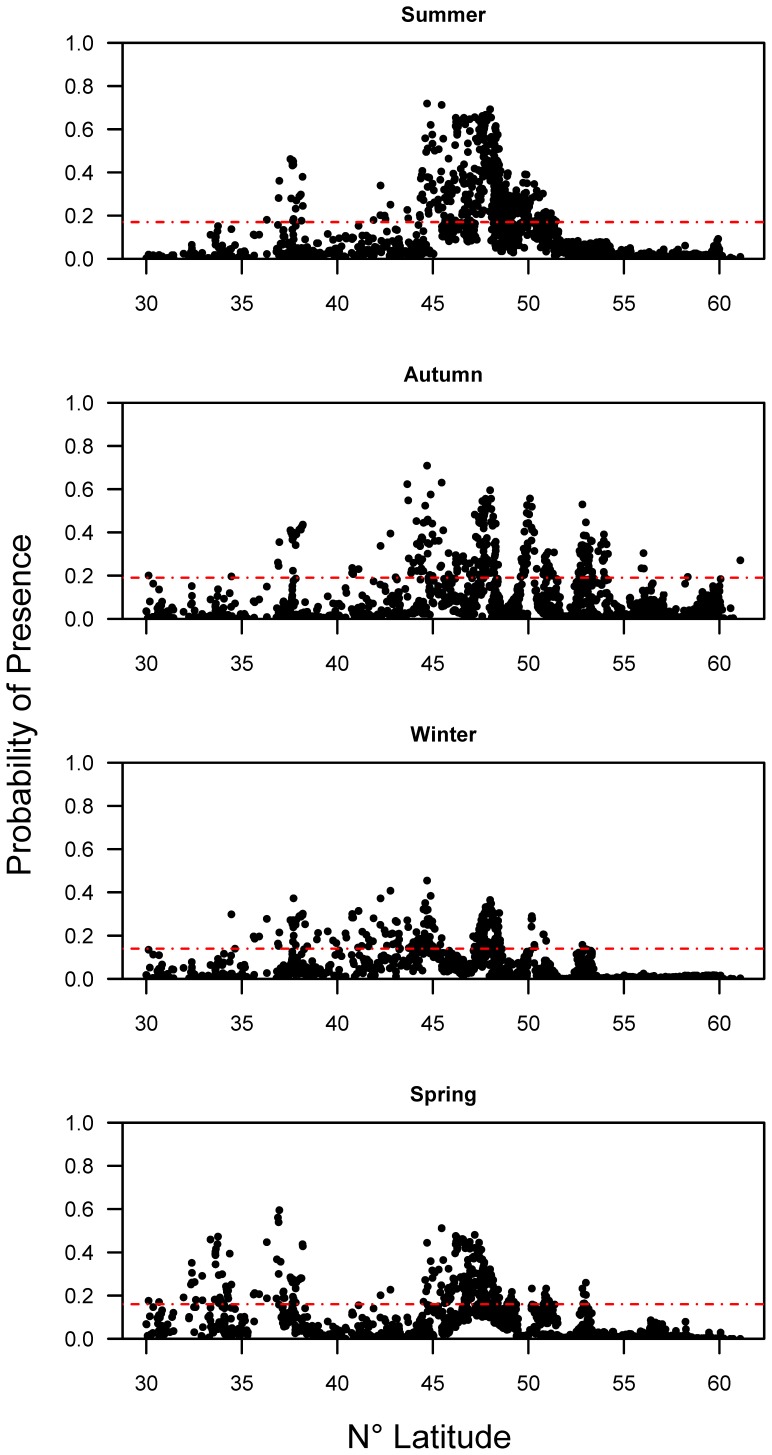
Seasonal probability of presence for MaxEnt background predictions. Each panel depicts the seasonal MaxEnt model predictions versus latitude for background samples. The red dashed line indicates the logistic presence-absence threshold value (described in the text) for each season.

The predicted probability of presence (logistic output) at all background sites (Table 1; prevalence) ranged from 0.03–0.05, indicating that sturgeon were predicted to occur in a relatively small proportion of their potential oceanic range at any given time. Our models predicted that green sturgeon presence would vary somewhat throughout the species range across seasons, but the primary concentration of sturgeon was estimated to be from approximately 41–51.5° N within the 200 m isobath along the west coast of North America (Figure 4). Notable concentrations were also predicted north of this region in the vicinity of the Queen Charlotte Islands (52–54° N), south near San Francisco Bay to south of Monterey Bay (35.5–38° N), and in the southern California Bight (33–34° N). We overlaid an independent fishery observer dataset from the west coast of the United States on our model probability map (Figure 4) to corroborate our model distribution results and found that sturgeon were captured in high probability areas. Additional details regarding this data set, including fishing effort and proportions of green sturgeon caught are displayed in Figure S3. There was a high degree of overlap in the seasonal predicted sturgeon distribution across latitudes (Figure 5, Figure S4). The summer distribution was highly concentrated between about 44° N and 51° N, with a secondary peak at about 38° N. The distribution spread out in the autumn and shifted north primarily from about 40–54° N, and in the winter it contracted slightly southward from 35–50° N. The spring distribution was the most southerly, and was bimodal; predicted presence peaked from 32–38° N and from 45–49° N.

### Latitudinal Distribution of Seasonal Covariates and Response Curve Thresholds

**Figure 6:**
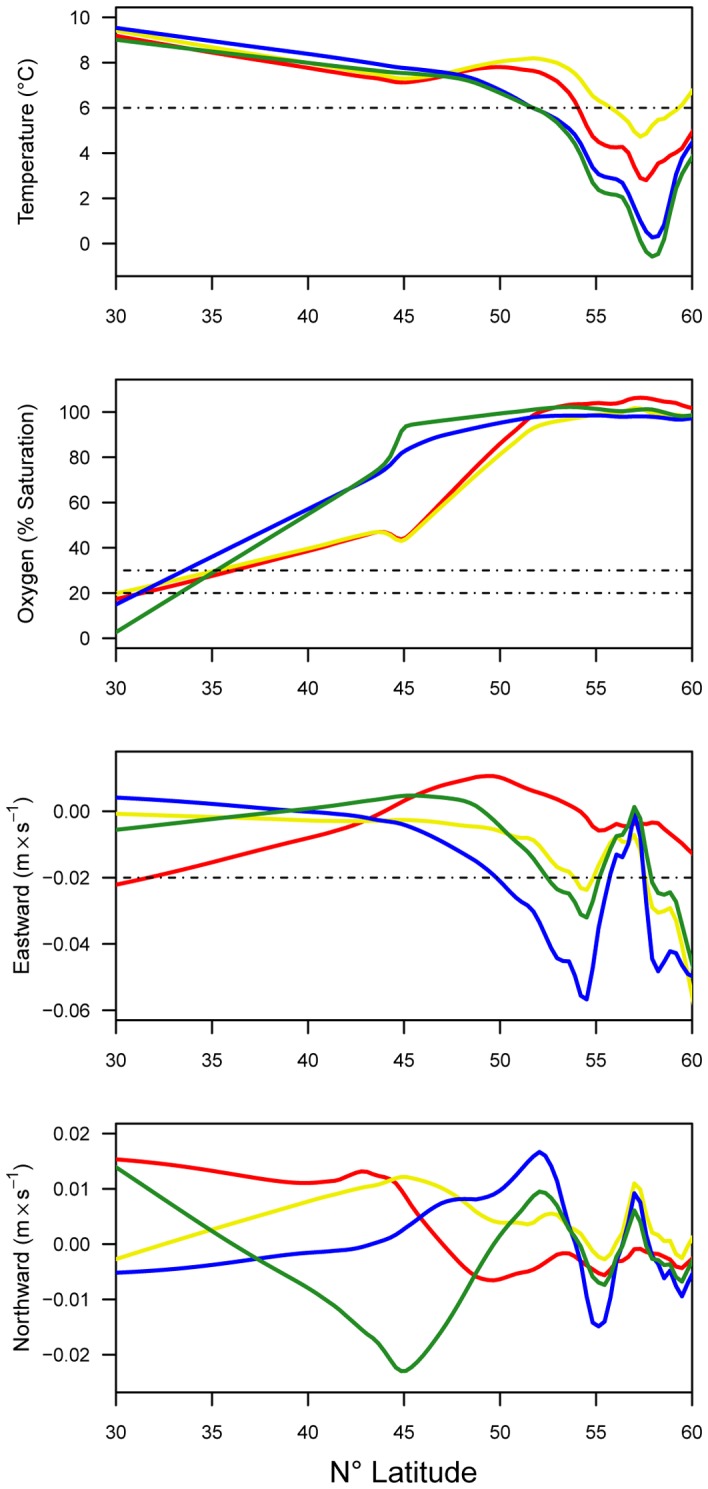
Seasonal model covariate values by latitude. Smoothed (loess) lines generated using model seasonal covariate background values across the green sturgeon range from 30 to 60° N; red = summer, yellow = autumn, blue = winter, green = spring. The dashed lines represent green sturgeon response threshold values that are described in the text (see Figure 2).

Across latitudes 30–60° N we compared sturgeon covariate suitability thresholds estimated from the response curves (Figure 2) with latitudinal seasonal covariate patterns (Figure 6) to elucidate potentially limiting factors affecting green sturgeon distribution. The latitudinal trend in temperatures was roughly similar across all seasons from 30–48° N; temperatures decreased from 9–10°C in the south to <8°C in the north. North of 51° N, winter and spring temperatures decreased sharply and remained lower than the MaxEnt threshold for sturgeon presence in spring and winter (>6°C) to the northern limit of their range. The dissolved oxygen presence-threshold was low (20% saturation in winter and spring and 30% in summer and autumn) relative to the distribution of dissolved oxygen values throughout the range (Figure 3) and only fell below the threshold in the extreme southern portion of the range near 30–33° N (Figure 6). The upper limit for eastward current (based on Figure 2) was 0.02 m sec^−1^ for autumn, winter and spring. During these seasons it only surpassed the modeled threshold during the winter from about 50–56° N and from 58–60° N and in the spring and autumn near 55° N and >58° N (Figure 6). Northward current appeared to be within the suitable range throughout the green sturgeon distribution in all seasons (Figure 6).

## Discussion

Although green sturgeon may be found as far north as the eastern Bering Sea (60° N) and as far south as the shores of the Baja Peninsula (30° N), their consistently inhabited range is much smaller. Our models predicted that green sturgeon presence varied somewhat seasonally, but the persistent concentration of sturgeon was estimated to be from approximately 41–51.5° N and in the vicinity of San Francisco and Monterey Bays from 36–37° N latitude. Trawl logbook records, fisheries observer records, and tagging studies support our inference that green sturgeon are primarily concentrated in the coastal waters of Washington, Oregon, and Vancouver Island [4], [5], [12]. An independent fisheries observer dataset [47] for the United States coast overlaid on our model probability map corroborated our distribution results by demonstrating that sturgeon were captured almost exclusively in high probability areas. Green sturgeon have also been recorded in modeled high probability areas in Canadian waters near Vancouver Island and Haida Gwaii (see Figure 6 in Lindley et al. 2008 [5]).

Green sturgeon are likely influenced by countervailing pressures that govern their dispersal. Their distribution is physiologically bounded by environmental features such as temperature, that control metabolic processes directly, or by factors that exact a significant metabolic toll, such as maintaining position in strong water currents or swimming and hiding to avoid predators. Yet, they are behaviorally directed to revisit natal freshwater spawning rivers every few years and to return to overwintering grounds in coastal marine habitats [48]. Marine ecosystems vary temporally, but there are areas of relatively stable and abundant food resources, often referred to as biological “hotspots,” that attract green sturgeon over great distances from their spawning rivers [5], [49]. For the purposes of our study, we considered the most prominent known sturgeon hotspot in the north to be a stable attractor for overwintering, just as fidelity to particular freshwater rivers in the south are deemed stable attractors for spawning [5], [50]. Across the wide geographic range of this highly mobile species, spawning areas and highly productive marine regions are persistent in space and time. Consequently, some of the most significant environmental variations to interact with behavioral drivers are broad-scale oceanographic features.

Beyond established species climatic or other environmental thresholds, individual presence records likely may not represent reproducing individuals and generally should not be included in species distribution models [51]. For green sturgeon, we were justified in including rare occurrences from the far extremes of their range in our models because well established, laboratory-derived physiological parameters were not available and green sturgeon are highly mobile. Rather, our understanding of temperature and depth preferences are based on statistically summarized field data which are limited in scope to relatively few archival tagged sturgeon [4], [24]. Statistical models such as MaxEnt may not be able to identify the extremes of sturgeon tolerance limits, especially given that we used data from acoustic receiver locations for studies that were not intended to examine sturgeon environmental bounds [52]. However, MaxEnt models have been shown to provide reasonable estimates of species distributions and accurate responses to environmental factors within a species’ known range [53]–[55]. Our MaxEnt models are not mechanistic, nor do they explicitly include biotic interactions. Nonetheless, they depict patterns and provide understanding of relevant oceanographic predictors that have a functional relationship with the biology of green sturgeon. Presence-only modeling applications such as MaxEnt provide a valuable alternative for utilizing opportunistically collected data for highly mobile species from sources such as acoustic arrays, in which valid absences may be difficult to obtain [56]. MaxEnt presence-only models may be particularly appropriate for highly dispersed fishes such as the green sturgeon, in combination with recently available oceanographic models, because these models are often primarily controlled by climactic regulators which are most appropriately analyzed at a coarse resolution and over a large extent [57].

Different suites of environmental features influenced sturgeon distribution models in each season. Although increasing temperatures and distance to southern spawning grounds are correlated, most sturgeon do not spawn in any given year, therefore spawning behavior may only be expected to describe a portion of the spring distribution [58]. Temperature may be an important limiting factor in the northern portion of the range. Temperature is often a key environmental gradient known to be a primary determinant of species distributions in aquatic habitats, often affecting fishes physiologically in one direction, and through biotic interactions in the other [59], [60]. For green sturgeon in the marine environment, low temperatures may be physiologically limiting [61]. Previous studies reported mean water temperatures inhabited by green sturgeon in the ocean to be >10°C, which is at the warm extremity of water temperature distribution throughout their range and concurs with the warmer temperature tendency for sturgeon in this study [4], [24]. Our modeled responses support unsuitably cold water temperatures, in addition to water current patterns (discussed below), as an explanation for why sturgeon are largely absent from the Bering Sea and areas north of the Queen Charlotte Islands. Fisheries records examined in Colway and Stevenson [12] indicated that the United States National Marine Fisheries Service database of Alaskan groundfish catches dating back to the 1960s and fisheries observer records from 1986 did not contain any records of green sturgeon, and few records have been reported in other databases from these waters [12], [62]. Therefore, it is most plausible that cold temperatures, perhaps in combination with other factors related to the perils of dispersing far from spawning grounds, is an important reason that green sturgeon are rare visitors north of 54°N latitude.

Current velocity and direction may also influence the green sturgeon’s putative tendency to migrate north after leaving bays and estuaries in the autumn, rather than turning south. Some evidence suggests that green sturgeon use currents to their energetic advantage when making long distance movements [63]. Green sturgeon are capable of swimming much faster than our modeled average bottom current velocities; typical migration speeds are about 40 km d^−1^ [5], whereas the maximum modeled current speeds where green sturgeon were observed was <10 km d^−1^. Nonetheless, green sturgeon migration patterns may be influenced by the relative ease of traveling north with the poleward surface expression of the California Undercurrent (or Davidson Current) in the autumn, rather than swimming south against it. The northern overwintering aggregation area coincides roughly with the end of the Davidson Current, and therefore, it may not be energetically favorable to travel farther north. Green sturgeon may then return to the southern bays, estuaries, and spawning rivers in the spring with the equatorward geostrophic flow brought on by the rapid strengthening of northwesterly winds, usually between March and May [20], [21], [64]. The California Undercurrent may also have been significantly faster during prolonged periods in the past when climate conditions enhanced it [65], [66], thus, the metabolic cost associated with swimming against the current may have had a greater effect on green sturgeon migratory behavior on an evolutionarily relevant timescale.

Given their temperature response, green sturgeon might be expected to have a more southerly distribution than our models predicted. Indeed, during model development we examined the predicted range of green sturgeon without an overwintering attractor covariate and found that the predicted distribution was substantially shifted southward in autumn and winter. A northward attraction to abundant food and refuge from predators in the topographically complex areas along the coast of Oregon, Washington, and Vancouver Island, provide alternative biotic explanations for the relatively sparse southerly green sturgeon distribution that our oceanographic or distance-based covariates could not elucidate [24]. Very little is known about green sturgeon feeding ecology in the ocean, but it is possible that a dissimilar suite of biotic influences south of Point Conception (34.4° N), which is a known faunal demarcation point for benthic macrofaunal assemblages and marine fishes [67], [68], decreases green sturgeon habitat suitability there. Dissolved oxygen may also play a role in constraining sturgeon habitat in the south extreme of the range. Physiologically, dissolved oxygen may be too low for green sturgeon in the extreme south, but we have so few presence records among low dissolved oxygen conditions that our results were inconclusive in this regard. However, dissolved oxygen is partly related to cycles of primary production and respiration, thus patterns in dissolved oxygen levels may reflect patterns of production in time and space. Although the complexity of these relationships could prove difficult to understand, an investigation of the indirect effects of dissolved oxygen on sturgeon distribution in the south could provide additional insight.

It may be difficult for sturgeon to find suitable habitat in the far southern or northern portions of their predicted distribution because it occurs in small patches and may be too isolated from other suitable areas. This may be especially true if there is too much intervening poor habitat along the central California and southern Alaskan coasts, as our models indicate. Vulnerability to predators may also discourage migration through areas with an abundance of predators such as sharks or pinnipeds which have been shown to prey on green sturgeon [69]–[71]. Most landings of green sturgeon as bycatch in the southern portion of their range appear to be from boats near San Francisco Bay, California; reports of green sturgeon from south of Monterey Bay to Point Conception, the southern California Bight, and the northern Baja Peninsula, Mexico are relatively scarce [11], [47], [72]. However, the status of green sturgeon presence in the far southern portion of the range is in need of further investigation. We are not aware of any formal green sturgeon studies from this region, and there are few reports that opportunistically address green sturgeon south of Monterey Bay, California [11], [62].

Highly migratory animals depend on functional migratory pathways to connect the variety of habitats that they require to complete their life cycle. Problems anywhere along the migration circuit can lead to population declines. It is perhaps not surprising that highly migratory species appear to be declining at faster rates than similar species that do not migrate as much [73]. Moreover, it is difficult for resource managers to identify the source of problems that might be distant from locations where the animals are routinely monitored. Seabed disturbances such as cable laying or activities such as bottom trawling may have negative impacts on benthic habitats by modifying substratum and altering soft-bottom communities and non-target fish assemblages. Areas closed to bottom trawling such as trawl rockfish conservation areas, marine sanctuaries, and other state waters may help to minimize green sturgeon bycatch, but sturgeon are still vulnerable in many areas where fishing occurs in both Canadian and United States waters [5], [62], [72]. These problems are exacerbated when animals move across jurisdictional boundaries, where regulatory regimes and threats may differ dramatically. Habitat models such as ours will help guide conservation efforts, such as marine spatial planning, by providing a better understanding of green sturgeon distribution across broad marine landscapes and may be used to estimate the impacts of predicted climatic variation. Risk evaluation for threatened species such as green sturgeon requires managers to consider the species’ spatial and temporal distribution and the relative carrying capacity of habitat in relation to human-influenced factors that may affect survival and abundance [1], [2]. The impact of management decisions or human activities on green sturgeon must be judged on the basis of the best scientific and commercial information that is available. Aside from harvest as bycatch in commercial and recreational fisheries, few other threats in the marine environment have previously been recognized [1], [2]. An improved understanding of important marine habitat will help to elucidate risks to green sturgeon that were formerly undetermined.

## Supporting Information

Figure S1 MaxEnt receiver operating curves (left) and plots of the omission rate for test model runs (right) as a fraction of background habitat predicted versus the cumulative threshold of suitable habitat.
(PDF)

Figure S2 Jackknife of test gain for the covariates. Gain is indicated by light blue bars without the variable, dark blue bars with the variable by itself, and the red bar indicates gain for the model with all variables. “U” represents eastward current and “V” represents northward current.
(PDF)

Figure S3 Distribution of green sturgeon bycatch in limited entry trawl and California halibut fisheries from 2002–2010 in United States waters along California, Oregon and Washington. Data are from the West Coast Groundfish Observer Program administered by the United States National Oceanic and Atmospheric Administration. The number of sets and number of green sturgeon per set are summarized per 10 km^2^. Green Sturgeon were captured at 269 out of 55711 sets. Specific vessel locations are not indicated on the map. The background layer is from the ESRI Ocean Basemap (2012). The map is displayed in the Albers Equal Area projection.
(PDF)

Figure S4 Seasonal predicted distribution maps. Background is the ESRI Ocean Basemap (2012). The map is displayed in Albers Equal Area Projection.
(ZIP)
