# Supplementary material for: Green Sturgeon Distribution in the Pacific Ocean Estimated from Modeled Oceanographic Features and Migration Behavior
Source: PLoS One. 2012 Sep 21;7(9):e45852. doi: 10.1371/journal.pone.0045852 (PMC3448713; doi:10.1371/journal.pone.0045852)
Supplement: Figure S1 — MaxEnt receiver operating curves (left) and plots of the omission rate for test model runs (right) as a fraction of background habitat predicted versus the cumulative threshold of suitable habitat. (PDF) [file pone.0045852.s001.pdf]

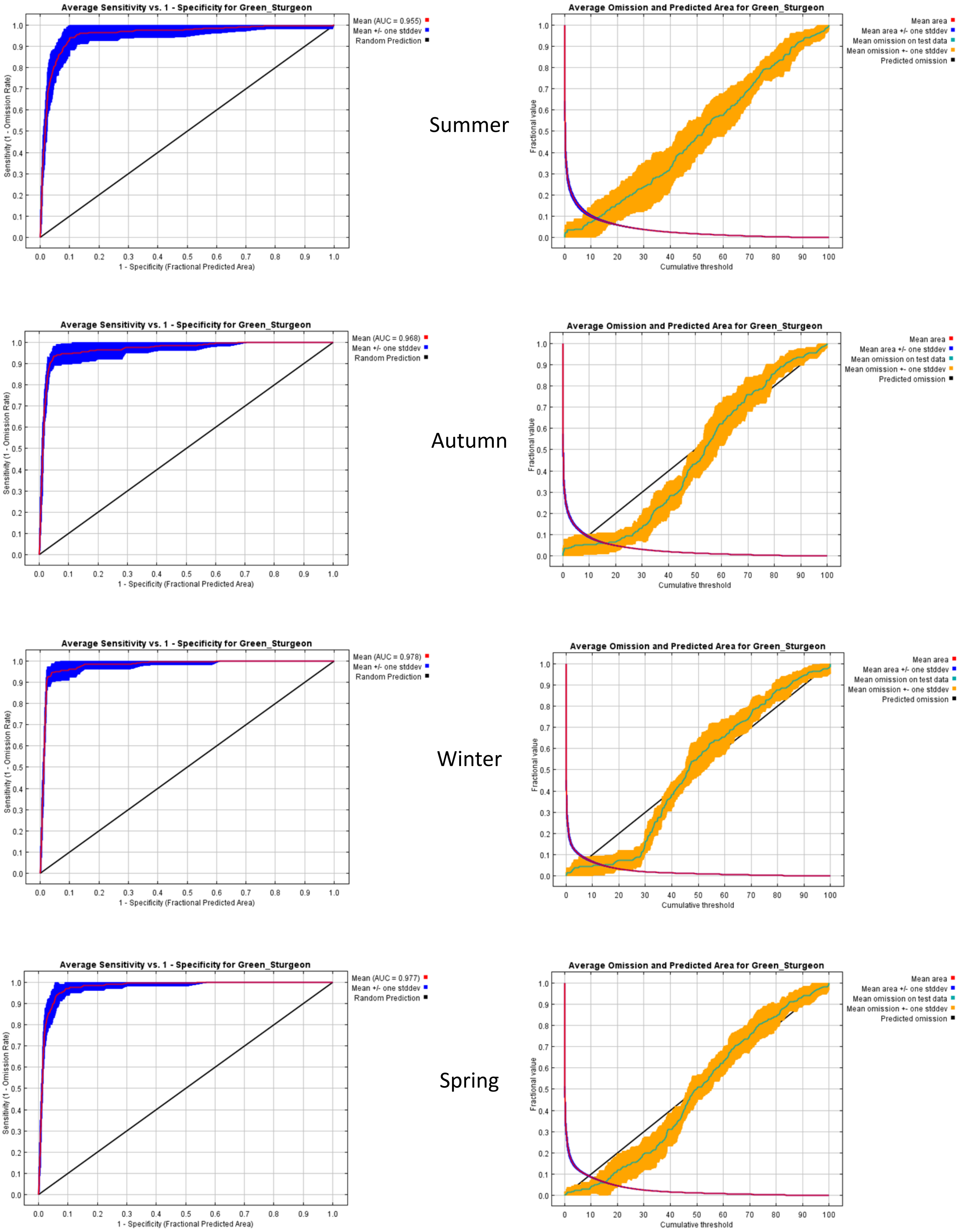

Figure S1. MaxEnt receiver operating curves (left) **and plots of the omission rate for test model runs** (right) as a fraction of background habitat predicted versus the cumulative threshold of suitable habitat.
