# Supplementary material for: Green Sturgeon Distribution in the Pacific Ocean Estimated from Modeled Oceanographic Features and Migration Behavior
Source: PLoS One. 2012 Sep 21;7(9):e45852. doi: 10.1371/journal.pone.0045852 (PMC3448713; doi:10.1371/journal.pone.0045852)
Supplement: Figure S2 — Jackknife of test gain for the covariates. Gain is indicated by light blue bars without the variable, dark blue bars with the variable by itself, and the red bar indicates gain for the model with all variables. “U” represents eastward current and “V” represents northward current. (PDF) [file pone.0045852.s002.pdf]

Summer

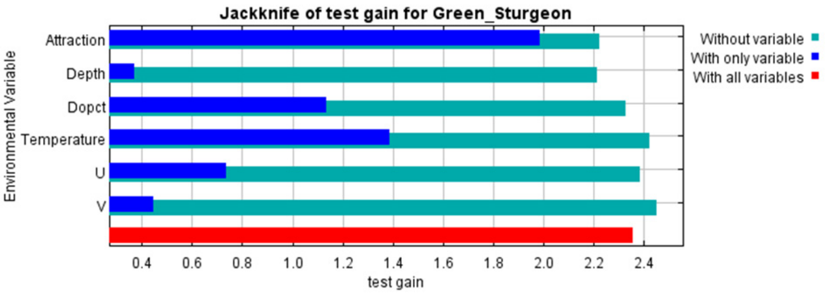

Autumn

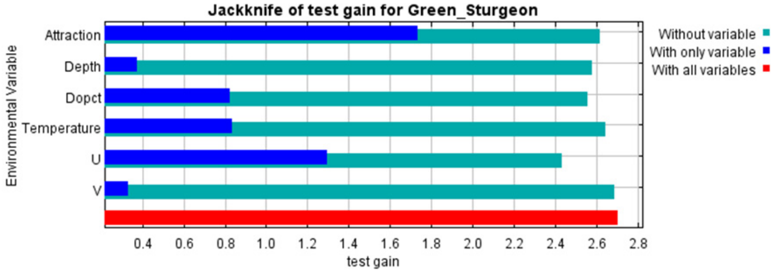

Winter

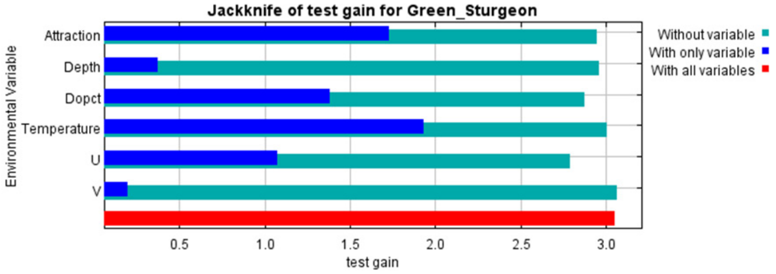

Spring

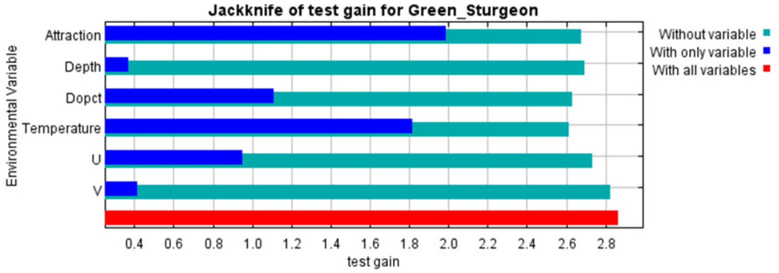

Figure S2. Jackknife of test gain for the covariates. Gain is indicated by light blue bars without the variable, dark blue bars with the variable by itself, and the red bar indicates gain for the model with all variables. “U” represents eastward current and “V” represents northward current.
