# Supplementary material for: Green Sturgeon Distribution in the Pacific Ocean Estimated from Modeled Oceanographic Features and Migration Behavior
Source: PLoS One. 2012 Sep 21;7(9):e45852. doi: 10.1371/journal.pone.0045852 (PMC3448713; doi:10.1371/journal.pone.0045852)
Supplement: Figure S3 — Distribution of green sturgeon bycatch in limited entry trawl and California halibut fisheries from 2002–2010 in United States waters along California, Oregon and Washington. Data are from the West Coast Groundfish Observer Program administered by the United States National Oceanic and Atmospheric Administration. The number of sets and number of green sturgeon per set are summarized per 10 km2. Green Sturgeon were captured at 269 out of 55711 sets. Specific vessel locations are not indicated on the map. The background layer is from the ESRI Ocean Basemap (2012). The map is displayed in the Albers Equal Area projection. (PDF) [file pone.0045852.s003.pdf]

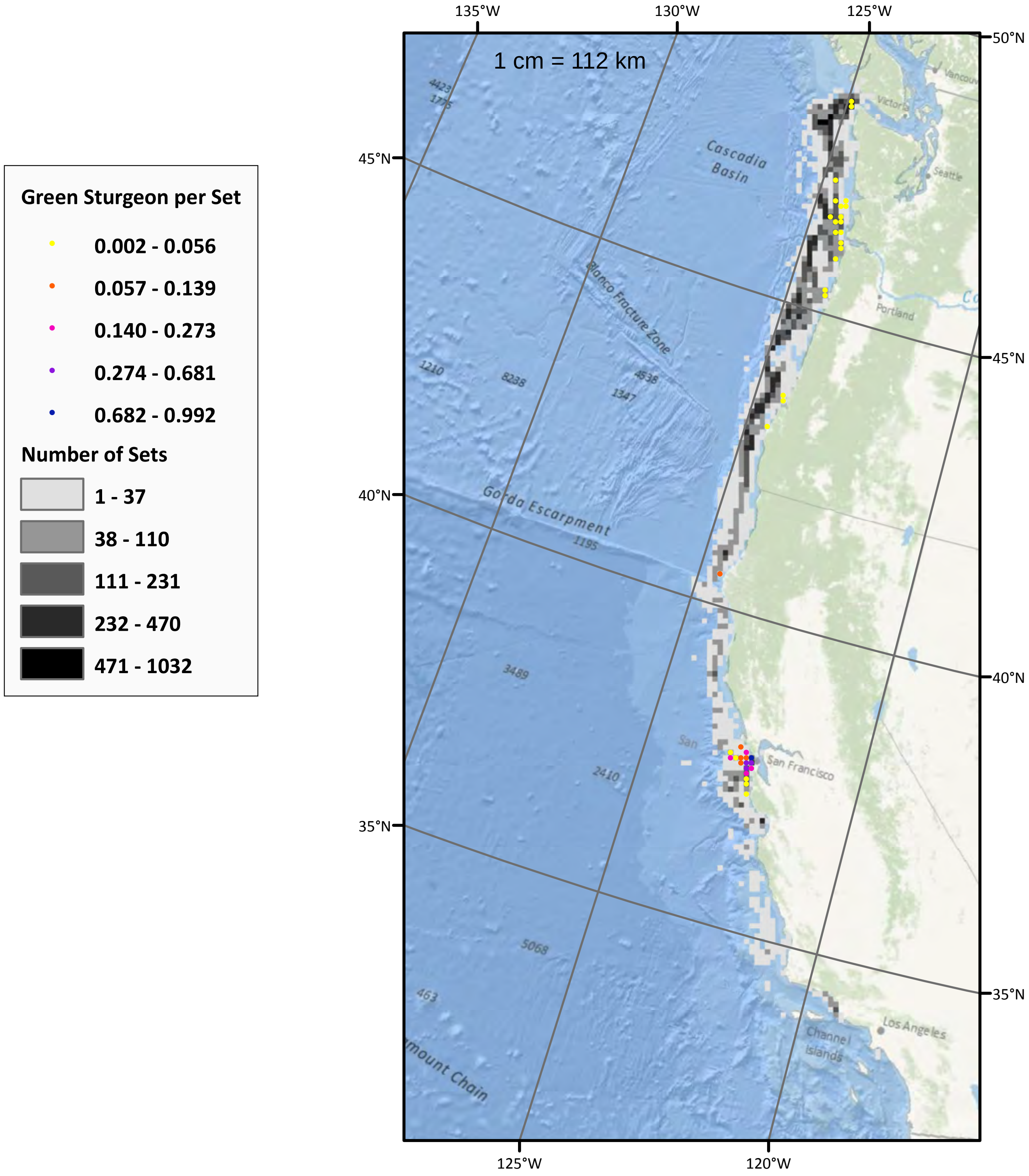


**Figure S3. Distribution of green sturgeon bycatch in limited entry trawl and California halibut fisheries from 2002-2010 in United States waters along California, Oregon and Washington.** Data are from the West Coast Groundfish Observer Program administered by the United States National Oceanic and Atmospheric Administration. The number of sets and number of green sturgeon per set are summarized per 10 km$^2$. Green Sturgeon were captured at 269 out of 55711 sets. Specific vessel locations are not indicated on the map. The background layer is from the ESRI Ocean Basemap (2012). The map is displayed in the Albers Equal Area projection.
