# Supplementary material for: Green Sturgeon Distribution in the Pacific Ocean Estimated from Modeled Oceanographic Features and Migration Behavior
Source: PLoS One. 2012 Sep 21;7(9):e45852. doi: 10.1371/journal.pone.0045852 (PMC3448713; doi:10.1371/journal.pone.0045852)
Supplement: Figure S4 — Seasonal predicted distribution maps. Background is the ESRI Ocean Basemap (2012). The map is displayed in Albers Equal Area Projection. (ZIP) [file pone.0045852.s004.zip › Figure S4.pdf]

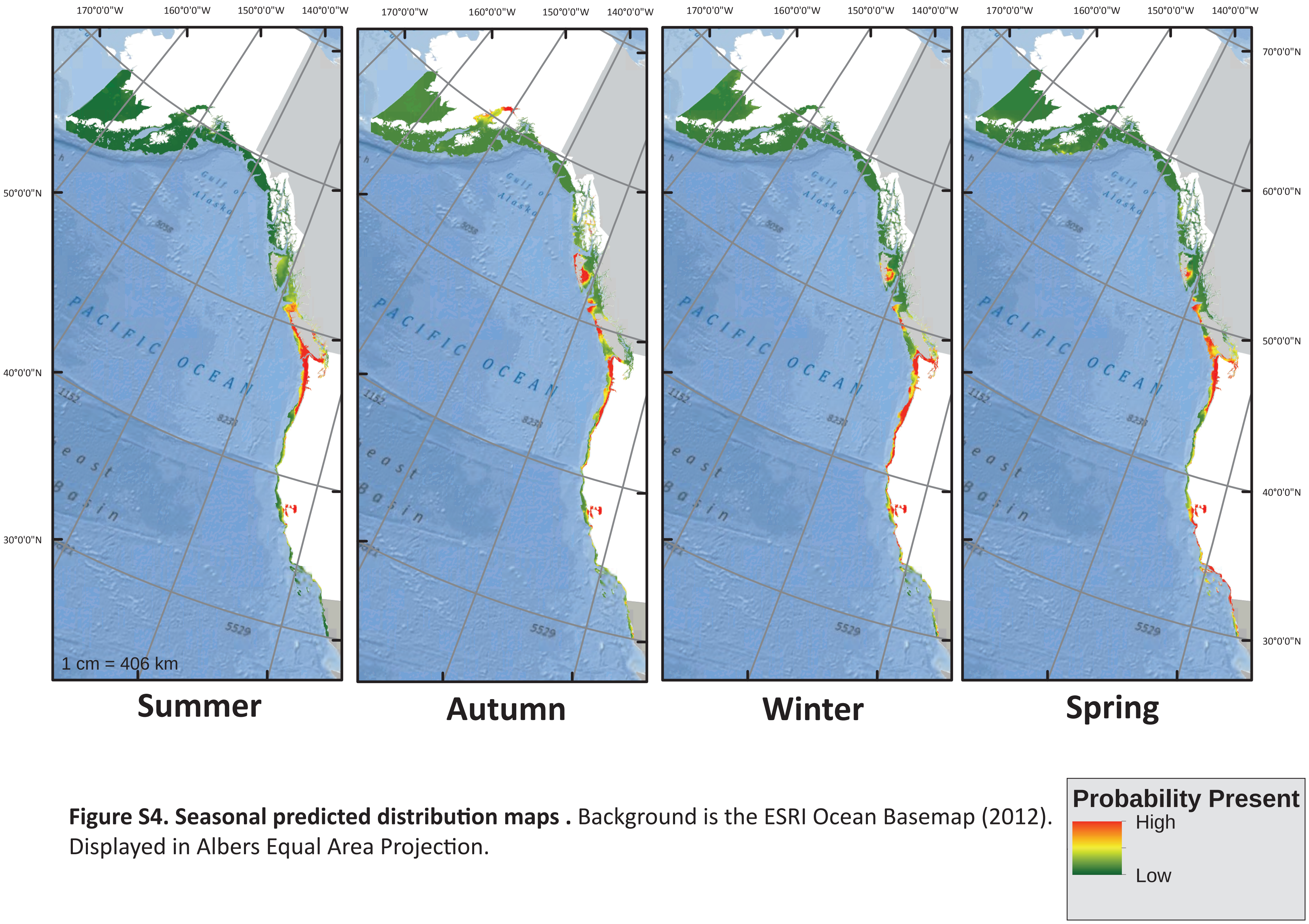

Figure S4. Seasonal predicted distribution maps . Background is the ESRI Ocean Basemap (2012). Displayed in Albers Equal Area Projection.
